# Postal methods for monitoring HbA1c in diabetes mellitus: a protocol for systematic review

**DOI:** 10.3399/BJGPO.2021.0240

**Published:** 2022-09-07

**Authors:** Jack Colley, Hajira Dambha-Miller, Beth Stuart, Jazz Bartholomew, Hermione Price

**Affiliations:** 1 Southern Health Foundation Trust, Southampton, UK; 2 Primary Care Research Centre, University of Southampton, Southampton, UK

**Keywords:** diabetes mellitus, glycated hemoglobin A, dried blood spot testing, primary health care

## Abstract

**Background:**

Worldwide there are an estimated 463 million people with diabetes. In the UK people with diabetes are offered annual review, which includes monitoring of haemoglobin A1c (HbA1c). This can identify people with diabetes who are not meeting their glycaemic targets, enabling early intervention. Those who do not attend these reviews often have poorer health outcomes. During the COVID-19 pandemic, there was a 77% reduction in monitoring of HbA1c in the UK.

**Aim:**

It is hypothesised that people with diabetes could take finger-prick samples at home for measurement of HbA1c. This study will examine the agreement and correlation of capillary HbA1c values compared with a venous reference standard. It will explore reliability and repeatability of capillary HbA1c testing methods, as well as the direction of effect of storage variables. The study will also explore patient acceptability and safety. It will look at capillary blood methods that would be suitable for posting.

**Design & setting:**

A systematic review will be undertaken.

**Method:**

The core terms of ‘Diabetes’, ‘HbA1c’ and ‘Capillary sampling’ will be used to search MEDLINE, Embase, Cumulative Index to Nursing and Allied Health Literature (CINAHL), Web of Science Core Collection, Google Scholar, OpenGrey, and other grey literature, from database inception until 2021. Risk of bias will be assessed using the ‘COSMIN Risk of Bias tool to assess the quality of studies on reliability and measurement error’.

**Conclusion:**

A narrative synthesis will be produced to explore whether there are viable postal alternatives to venous sampling, as well as exploring acceptability and safety of patient self-collection.

## How this fits in

Worldwide there are an estimated 463 million people with diabetes.^1^ In the UK people with diabetes are offered annual review which includes monitoring of Haemoglobin A1c (HbA1c).^2,3^ This can identify people with diabetes who are not meeting their glycaemic targets, enabling early intervention. Those who do not attend these reviews often have poorer health outcomes.^4^ During the COVID-19 pandemic, there was a 77% reduction in monitoring of HbA1c in the UK.^5^ The systematic review outlined in this protocol will explore the feasibility of patients taking finger-prick samples at home and posting these to a laboratory to obtain an HbA1c result.

## Introduction

Diabetes is one of the biggest health issues worldwide. Globally, there are an estimated 463 million people with the disease, a number that has increased four-fold over the past 35 years.^1,2,5^ In the UK, 4.9 million people are currently living with diabetes.^6^ The prevalence of both type 1 and type 2 diabetes is increasing; however, this rise is far more alarming in cases of type 2 diabetes.^7,8^

The diabetes annual review process consists of a series of health checks recommended by the National Service Framework and the National Institute for Health and Care Excellence (NICE).^3,9^ These health checks include monitoring of HbA1c, blood pressure, cholesterol, kidney function, urinary albumin, body mass index (BMI), and foot health. There is a financial incentive for practices that record a high percentage of patients achieving recommended treatment goals for HbA1c, blood pressure, and cholesterol. This process aims to improve standards of care by identifying where people with diabetes may not be meeting recommended targets.

During the COVID-19 pandemic, the introduction of social distancing led to many healthcare practitioners working remotely for delivery of routine care. People with diabetes were identified as clinically vulnerable and were advised to adhere stringently to social distancing guidance.^10–12^ As a consequence, in April 2020 there was a reported 77% reduction in monitoring of HbA1c in England. There was an estimated 60 000 missed or delayed new diagnoses of diabetes across the UK.^13^

Before the COVID-19 pandemic, non-attendance at diabetes outpatient appointments was a sizeable problem. In England in the 2019–2020 financial year, over 6% of all appointments were not attended; rates are similar in people with diabetes.^14,15^ Perhaps more concerningly, a recent systematic review suggested non-attenders on average had higher HbA1c levels and worse health outcomes.^4^ A system of required attendance at a healthcare setting in order to measure venous HbA1c will inevitably fail to fully address this cohort, who arguably are the most in need of diabetes surveillance.

The authors hypothesise that people with diabetes could take finger-prick samples at home and post these to a laboratory to obtain an HbA1c result. There is a growing body of evidence surrounding the use of capillary blood, and dried blood spots in particular, for measuring HbA1c.^16–23^ This review will explore how well HbA1c values obtained from capillary blood, and stored via methods suitable for posting, agree and correlate with venous HbA1c results. It will also explore the reliability and repeatability of capillary HbA1c testing. There is also a need for standardisation of collection, storage, and transportation methods.^16,21,23^ This review will explore the direction of effect of storage variables on capillary HbA1c results.

It also needs to be ascertained whether a self-collected HbA1c test would be acceptable to patients, and what might influence the uptake of such a test. Finger-pricking itself is a regularly used method of blood collection in many patients with diabetes. However, it needs to be ascertained whether changes such as larger lancets for the collection of larger blood volumes, and their use in patients naïve to finger-pricking, may introduce previously unanticipated safety concerns.

This systematic review aims to explore all currently available evidence examining HbA1c measured from capillary blood compared with venous results, and whether there are viable postal alternatives to venous sampling. The evidence produced could be a step towards transforming current diabetes practices, especially in more remote communities. The COVID-19 pandemic has presented a clear and urgent need for improving access to health care remotely in people with diabetes.

### Objectives

#### Primary objectives

To determine:

1. Concurrent criterion validity: agreement and correlation of capillary HbA1c values with the reference standard of venous HbA1c values within subjects.
2. Reliability: variability of repeated HbA1c values within subjects.
3. The direction of effect of storage variables on HbA1c value, concurrent criterion validity, and reliability in capillary samples.

#### Secondary objectives

To answer the following questions:

- Is self-collected finger-prick sampling at home, as a means of measuring HbA1c, acceptable to people with diabetes as an alternative to venous blood sampling in a healthcare setting? What is the reported ease of use of self-collection equipment and what method of HbA1c monitoring would patients prefer?
- Can HbA1c be measured safely at home by people with diabetes mellitus? Are there any reported safety concerns regarding the use of capillary blood collection for the purpose of determining HbA1c?

### Study criteria

#### Population

Studies of adults aged ≥18 years who are being screened for, monitored, or previously diagnosed with diabetes mellitus. Studies that exclusively look at HbA1c measurement in people with haemoglobinopathies, anaemias, high erythrocyte turnover or other conditions known to affect HbA1c interpretation may be discussed but not included in analysis of validity. Animal studies will not be included.

#### Inclusion criteria and index test

Articles that examine methods of capillary blood self-collection and storage for measurement of HbA1c, which look at agreement or correlation with venous HbA1c results, reliability, repeatability, and patient acceptability. Only studies of methods that would be practical to post to people with diabetes will be included, thus excluding most point-of-care methods or those requiring high-intensity storage such as refrigeration. Studies of novel methods still in experimental stages, where validation studies with patient blood specimens have not been carried out, will not be included. Studies using venous blood to test novel methods for capillary blood collection will be included (for example, the use of venous blood on a dried blood spot). These studies will be differentiated from capillary studies in the analysis. Studies that look at the effect of time, temperature, and other storage variables on capillary HbA1c results will be included.

#### Target condition

The target condition is any form of diabetes mellitus where HbA1c would be used for diagnosis or monitoring. Routine use of HbA1c is not recommended in women in the second or third trimester of pregnancy and therefore studies in this cohort will not be included.^24^

#### Reference standard

For studies of accuracy or precision, the reference standard must be a venous blood test analysed by a certified method for HbA1c testing, as listed by the International Federation of Clinical Chemistry and Laboratory Medicine (IFCC) or the National Glycohemoglobin Standardization Program (NGSP).^25,26^ Studies will be excluded where the time interval between index test and reference standard is >2 weeks for >10% of participants.

## Method

This protocol is reported according to the Preferred Reporting Items for Systematic Reviews and Meta-Analyses (PRISMA) guidance and has been registered on PROSPERO.^27,28^

### Search strategy

The key search terms are ‘Diabetes’ AND ‘HbA1c’ AND ‘Capillary sampling’, which have been expanded in the full search strategy (Supplementary Box S1). Embase, MEDLINE, CINAHL and Web of Science Core Collection databases will be searched from database inception to September 2021. Additionally, grey literature will be searched through OpenGrey as well as using Google Scholar limiting retrieval to the first 200 relevant references as per guidance on optimal database combinations.^29^ Ongoing studies and other grey literature will be sought from relevant conference abstracts from 2020 and 2021. Reference lists from the most relevant articles will be hand-searched using an ancestry approach. Results will be restricted to those available in the English language only.

#### Study selection and data management

Two reviewers (JC and JB) will independently screen titles and abstracts against inclusion and exclusion criteria while blinded to each other’s decisions. Selected studies will then undergo full-text review by both reviewers. Any discrepancies that arise during this process will be discussed between the two reviewers until a resolution is reached. Where a resolution cannot be found, a third reviewer will be consulted. A PRISMA flow diagram will be used to demonstrate study selection and exclusion. Studies that undergo full-text review will be documented, and, if excluded at this stage, a record of the reasons for exclusion will be kept and this information will be made available.

A data extraction tool has been created using Microsoft Excel (Version 2102), which was piloted (Supplementary Table S1). Two reviewers will carry out data extraction and disagreements will be resolved through discussion.

The data being collected will include information regarding:

- Study characteristics (country; year; setting; objectives; and inclusion and exclusion criteria).
- Participant characteristics (population description; age; sex; and diabetic status).
- The capillary blood method or methods being investigated (including details about method used; person collecting the sample; location; storage and shipping conditions; laboratory methods including analysis device and timing between test and reference).
- The reference standard (including analysis device used; location of collection; storage and shipping conditions; laboratory blinding; and number of available results).
- The primary outcome (including statistical methods used and outcomes relevant to that method; measures of error and bias; and any other reported results).
- Additional outcomes (patient acceptability; and, where evidence exists to do so, information on facilitators and barriers to patient uptake).

#### Quality assessment

The study will use ‘COnsensus-based Standards for the selection of health Measurement INstruments (COSMIN) Risk of Bias tool to assess the quality of studies on reliability and measurement error of outcome measurement instruments’ to assess the quality of selected studies.^30,31^ The quality assessment will be conducted by two reviewers, any discrepancies that arise during this process will be discussed between the two reviewers until a resolution is reached. As a narrative synthesis is being performed, there will be no limit on what level of quality leads to inclusion. Instead, quality of studies will be discussed as part of the synthesis of their results.

### Data synthesis

From preliminary searches the authors have identified methodological heterogeneity in the studies identified. There is variability in how data have been presented: studies have either presented outcomes in terms of sensitivity and specificity of HbA1c as a diagnostic test; or at pre-defined clinically significant levels; or as measures of error, with correlation coefficients, intercepts, Bland-Altman plots, or error grid analyses. There is also heterogeneity in the collection, storage and transportation methods, and type of blood used for the index test. Full exploration of heterogeneity will take place following completion of searches, by visual examination of tables ordered by likely modifiers. Identified modifiers will be used to determine subgroups for synthesis, which will in turn be used to determine how these variables can impact validity.

It is anticipated the most appropriate method of synthesis will be narrative. The Synthesis Without Meta-Analysis (SWiM) reporting guidelines adapted for a non-interventional review will be followed.^32^ The SWiM guidance is tailored towards reviews of intervention, and therefore it may need adapting as the synthesis is undertaken. Where significant heterogeneity exists, meta-analysis can often fail to produce meaningful results by under-representing the overall body of evidence. Narrative synthesis will enable the authors to incorporate all available evidence despite heterogeneity.

Measurement outcomes and the appropriateness of each statistical method used to the study design will be discussed. Correlation and agreement outcomes will be summarised across all identified studies and vote counting will be used to inform the conclusions.

For the secondary outcome of patient acceptability, the method of synthesis will depend largely on the amount of data available. If evidence for patient acceptability is limited, uptake and dropout rates will be looked at, and this information will be used to infer probable acceptability of a proposed at-home collection option.

### Grading the strength of recommendations

The Grading of Recommendations, Assessment, Development, and Evaluations framework (GRADE) will be used in making the recommendations.^33^

## Discussion

### Summary

To the authors' knowledge, this is the first systematic review to explore all postal methods of capillary blood collection for the measurement of HbA1c.

### Strengths and limitations

Owing to anticipated heterogeneity in statistical approaches, summary analyses, sample storage, transportation, extraction, and assay, meta-analysis is unlikely to be appropriate and therefore narrative synthesis will be used.

### Implications for research and practice

This review will be submitted for publication in a peer-reviewed, open access journal. Where invited to do so, the results will be presented at both national and international relevant conferences. These methods will allow dissemination to patient groups, clinicians, and guideline developers.

The findings could help improve care for people with diabetes allowing home monitoring for those unable to attend clinics. This would enable early detection and intervention for changes in glycaemic control.

## Trial registration number

PROSPERO registration number CRD42021225606
